# Metabolic Fate of Fructose Ingested with and without Glucose in a Mixed Meal

**DOI:** 10.3390/nu6072632

**Published:** 2014-07-15

**Authors:** Fanny Theytaz, Sara de Giorgi, Leanne Hodson, Nathalie Stefanoni, Valentine Rey, Philippe Schneiter, Vittorio Giusti, Luc Tappy

**Affiliations:** 1 Department of Physiology, University of Lausanne, rue du Bugnon 7, CH-1005 Lausanne, Switzerland; E-Mails: fanny.theytaz@unil.ch (F.T.); sara.degiorgi@hibroye.ch (S.G.); nathalie.stefanoni@unil.ch (N.S.); valentine.rey@unil.ch (V.R.); philippe.schneiter@unil.ch (P.S.); 2 Oxford Centre for Diabetes, Endocrinology and Metabolism, University of Oxford, Oxford OX3 7LE, UK; E-Mail: leanne.hodson@ocdem.ox.ac.uk; 3 Service of Endocrinology, Diabetes and Metabolism, Lausanne University Hospital, CH-1011 Lausanne, Switzerland; E-Mail: vittorio.giusti@hibroye.ch

**Keywords:** fructose oxidation, gluconeogenesis, glucose production, *de novo* lipogenesis, hepatic, intestinal, sugar

## Abstract

Ingestion of pure fructose stimulates *de novo* lipogenesis and gluconeogenesis. This may however not be relevant to typical nutritional situations, where fructose is invariably ingested with glucose. We therefore assessed the metabolic fate of fructose incorporated in a mixed meal without or with glucose in eight healthy volunteers. Each participant was studied over six hours after the ingestion of liquid meals containing either ^13^C-labelled fructose, unlabeled glucose, lipids and protein (Fr + G) or ^13^C-labelled fructose, lipids and protein, but without glucose (Fr), or protein and lipids alone (ProLip). After Fr + G, plasma ^13^C-glucose production accounted for 19.0% ± 1.5% and ^13^CO2 production for 32.2% ± 1.3% of ^13^C-fructose carbons. After Fr, ^13^C-glucose production (26.5% ± 1.4%) and ^13^CO_2_ production (36.6% ± 1.9%) were higher (*p* < 0.05) than with Fr + G. ^13^C-lactate concentration and very low density lipoprotein VLDL ^13^C-palmitate concentrations increased to the same extent with Fr + G and Fr, while chylomicron ^13^C-palmitate tended to increase more with Fr + G. These data indicate that gluconeogenesis, lactic acid production and both intestinal and hepatic *de novo* lipogenesis contributed to the disposal of fructose carbons ingested together with a mixed meal. Co-ingestion of glucose decreased fructose oxidation and gluconeogenesis and tended to increase ^13^C-pamitate concentration in gut-derived chylomicrons, but not in hepatic-borne VLDL-triacylglycerol (TG). This trial was approved by clinicaltrial. gov. Identifier is NCT01792089.

## 1. Introduction

Fructose intake has increased progressively over the past 30 years and currently averages about 50–60 g/day in the U.S. and in several European countries [1,2]. There is much concern that this may contribute to the rise in prevalence of obesity and metabolic disorders observed over the same period [3]. This is mainly based on the observation that a high-fructose-containing diet causes obesity, diabetes mellitus, dyslipidemia and hepatic steatosis in rodents [4,5] and that short-term fructose overfeeding increases fasting and postprandial triacylglycerol (TG), decreases hepatic insulin sensitivity and increases intrahepatic fat in humans.

Several short-term studies, using ^13^C-fructose as a metabolic tracer, have documented that fructose exerts specific metabolic effects, which may indeed contribute to the pathogenesis of insulin resistance and dyslipidemia. Fructose is largely extracted by splanchnic organs (gut and liver), where it is essentially converted into glucose, lactate and fatty acid, which can subsequently be used as an energy substrate by extrahepatic cells. After the administration of a large ^13^C-labelled fructose load, within 4–6 h, approximately 50% of the labelled carbons are recovered as breath ^13^CO_2_, indicating that 50% are temporarily retained in the body’s energy stores [6,7,8]; over the same period, about 30%–50% is released in the blood as ^13^C-labelled glucose [6,7,8] and an unspecified amount is released as ^13^C-labelled lactate. The oxidation of these newly synthesized substrates obviously contributes to total breath ^13^CO_2_ production, but the relative amounts of ^13^C glucose being immediately oxidized in extra-hepatic tissues and stored as extra-hepatic (mainly muscle) glycogen is not known. After intravenous administration, hepatic and muscle glycogen synthesis account each for about 25% of total fructose infused [9], but similar data are not available for oral fructose administration. Labelled carbon atoms are also recovered as very low density lipoprotein VLDL-fatty acids, indicating that hepatic *de novo* lipogenesis is active after fructose ingestion [10]. Tracer studies using ^13^C-acetate as a lipogenic precursor further indicate that fructose is substantially more lipogenic than glucose [11]. *De novo* lipogenesis is generally assumed to occur in the liver, but may also take place in the gut [12,13]. It is enhanced by the consumption of a high fructose diet [14], and contributes, together with a decrease of TG-rich lipoprotein clearance [15,16], to fructose-induced hypertriglyceridemia.

The relevance of studies assessing the effects of pure fructose on the pathogenesis of metabolic diseases is often questioned, as dietary fructose is mainly present in fructose-containing caloric sweeteners (sucrose, high fructose corn syrup), fruits and honey, which all contain roughly equimolar amounts of fructose and glucose. Co-ingestion of glucose or other nutrients may indeed significantly alter the metabolic fate of fructose due to changes in glucoregulatory hormone concentrations. In addition, glucose and fructose are expected to mutually enhance their splanchnic metabolism, since the presence of glucose in the gut lumen increases intestinal fructose absorption, on the one hand [17], and fructose-1-P activates hepatic glucokinase, resulting in hepatic glucose metabolism and glycogen storage, on the other hand [18]. The aim of the present study was to assess the metabolic fate of fructose and its interaction with other nutrients present in a mixed meal. For this purpose, we monitored, in healthy human volunteers, the metabolic response to a liquid meal containing protein, fat, glucose and ^13^C-labelled fructose and to the same liquid meal in which glucose only or both glucose and fructose were omitted. These measurements were performed after subjects had consumed a weight-maintenance diet containing 20% sucrose for three days.

## 2. Experimental Section

### 2.1. Subjects Inclusion

Eight healthy non obese volunteers (4 males, 4 females) with a mean (±SEM) age and BMI of 26.4 ± 1.0 year and 21.9 ± 0.7 kg/m^2^, respectively, were included in a randomized, crossover study. Participant characteristics are shown in Table 1. All subjects were non-smokers, had low habitual physical activity, were not currently taking any medication and had no family history of diabetes. Before inclusion, they underwent a physical examination to ensure good physical health. The experimental protocol was approved by the Ethical Committee of Lausanne University School of Medicine. All participants provided written informed consent.

**Table 1:** Baseline characteristics of the studied participants (mean ± SD).

	Mean ± SD
Age (year)	26.4 ± 1.0
Body weight (kg)	63.7 ± 2.4
Body mass index (kg/m^2^)	21.9 ± 0.7
Body fat (%)	20.6 ± 1.9
Systolic blood pressure (mmHg)	118 ± 3
Diastolic blood pressure (mmHg)	70 ± 3
Heart rate (beats/min)	74 ± 3

### 2.2. Study Design

Each volunteer was studied on three different occasions according to a randomized cross-over design. On each occasion, participants first consumed a controlled weight-maintenance diet (basal energy requirements calculated with the Harris-Benedict equation times a physical activity factor of 1.5) containing 55% carbohydrate (35% complex carbohydrate and 20% sugar), 15% protein and 30% fat during 3 days. During this period, they were asked to have minimal physical activity and to abstain from alcohol or caffeine-containing beverages. On the fourth day, subjects came to the Clinical Research Center of Lausanne University Hospital at 7:00 am in the fasting state and underwent a metabolic test with the ingestion of 1 of the 3 following test meals:

Fr + G (glucose) meal: a liquid meal containing 7.99 ± 0.14 kcal/kg, 0.3 g/kg lipid (from cream), 0.3 g/kg protein (from Whey Protein 94, Sponser, Wollerau, Switzerland), 0.5 g/kg glucose (in 2 mL water) and 0.5 g/kg fructose (in 2 mL water). Fructose was labelled with 1% U-^13^C_6_-fructose (Cambridge Isotope Laboratories, Tewksbury, MA, USA). This meal accounted for 30% of their daily energy needs (calculated as basal energy requirements times 1.1 to account for very low physical activity during the test and for an expected thermic effect of food equal to 10% of the total energy requirements).

Fr (fructose) meal: a liquid meal containing 5.99 ± 0.09 kcal/kg, 0.3 g/kg lipid (from cream), 0.3 g/kg protein (from Whey Protein 94, Sponser, Wollerau, Switzerland) and 0.5 g/kg fructose (in 2 mL water). Fructose was labelled with 1% U-^13^C_6_-fructose. This meal accounted for 22% of the daily energy needs.

ProLip (protein and lipid) meal: a liquid meal containing 4.01 ± 0.06 kcal/kg, 0.3 g/kg lipid (from cream) and 0.3 g/kg protein (from Whey Protein 94, Sponser, Wollerau, Switzerland). This meal accounted for 15% of the daily energy needs. 

The order of administration of the three meals was randomized, and a washout period of 3–10 weeks was allowed between metabolic tests.

### 2.3. Metabolic Tests

**Figure 1:**
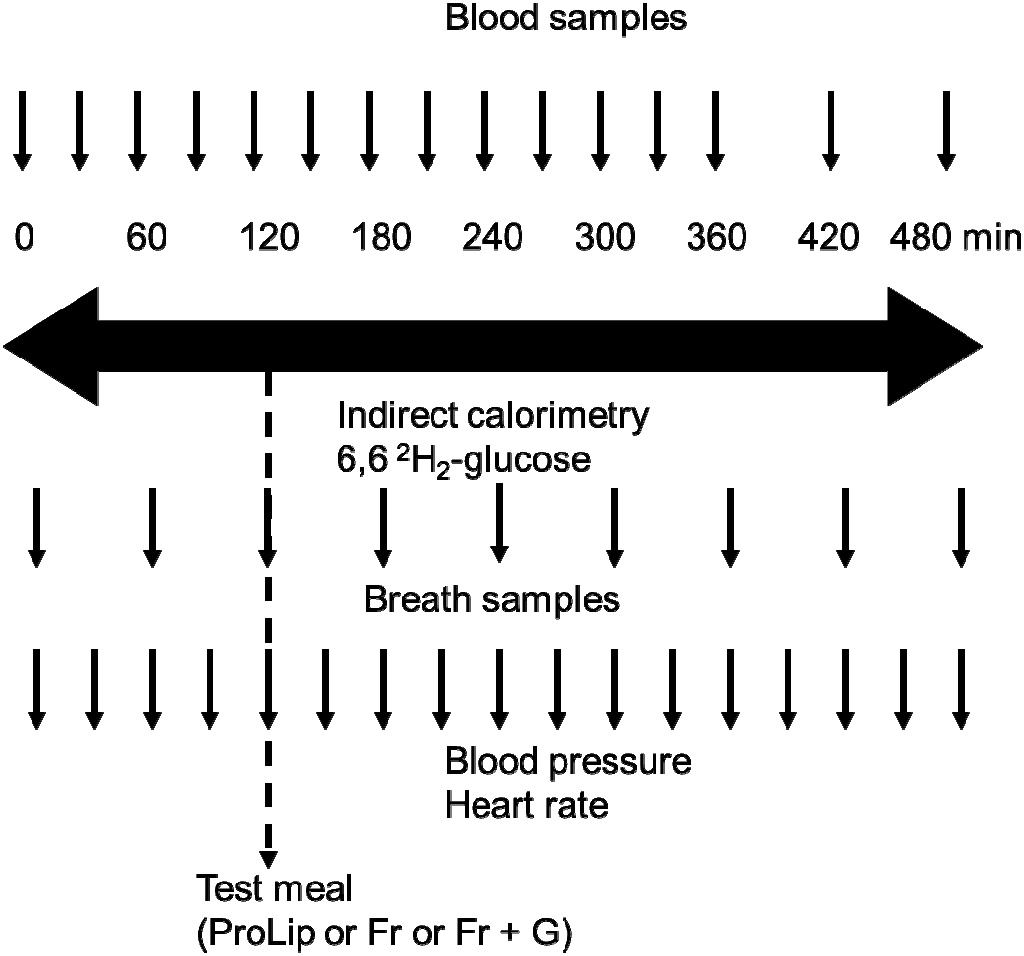
Graphical representation of metabolic tests. ProLip, protein and lipid; Fr, fructose; G, glucose.

Each metabolic test included 2-h fasting and 6-h postprandial measurements, as depicted in Figure 1.

On arrival, subjects were weighed and the body composition assessed by bio-electrical impedance (Imp Df 50; ImpediMed, Pinkenba, Australia). Subjects were asked to void their bladder, and the collected urine was discarded, with all urine thereafter collected until the end for the determination of the urinary urea nitrogen excretion rate. Subjects were then transferred to a bed, where they remained quiet, but awake for the next eight hours. A venous catheter was inserted into a forearm vein and was used for blood sampling. A second catheter was inserted into an antecubital vein of the other arm and a primed-continuous infusion of tracer amounts of 6,6-^2^H_2_-glucose (Cambridge Isotope Laboratories, Cambridge, MA, U.S.; bolus 2.8 mg/kg, continuous infusion 40 μg/kg/min) was administered through this catheter throughout the metabolic test to calculate whole body glucose rates of appearance (GRa) and of disappearance (GRd) [7,8]. Respiratory gas exchange was monitored throughout the experiment by open circuit indirect calorimetry (Quark RMR, version 9.1b, Cosmed, Rome, Italy). Energy expenditure and net substrate oxidation were calculated using the equations of Livesey and Elia [19]. Due to technical problems, results could not be obtained for 2 subjects during one of the three meals. Data collected during the other two meals in these two subjects were not taken into consideration to avoid introducing bias in paired statistical analyses. Blood samples were collected at Time 0 immediately before starting the 6,6-^2^H_2_-glucose administration (T = 0 min) and after two hours spent in fasting conditions (T = 120 min); thereafter, subjects consumed their test meal over a 15-min period (T = 120 min), during which indirect calorimetry was interrupted and blood samples were collected at T = 150, 180, 210, 240, 270, 300, 360, 420, 480 min. Hormones and metabolites, plasma 6,6-^2^H_2_-glucose, ^13^C-lactate and ^13^C-glucose enrichments and VLDL- and chylomicron-TG concentration and ^13^C-palmitate enrichment in chylomicron and VLDL subfractions on each blood sample were measured. Breath samples were collected for the measurement of ^13^CO_2_ isotopic enrichment at T = 0, 60, 120, 180, 240, 300, 360, 420 and 480 min when subjects had the Fr and Fr + G meals, and at T = 0, 120, 240, 360 and 380 min when they had the ProLip meal.

### 2.4. Analytical Procedures

Plasma was immediately separated from blood cells by centrifugation at 1230× *g* for 10 min at 4 °C, and plasma aliquots were stored at −20 °C. Plasma metabolites (glucose, TG, non-esterified fatty acids (NEFAs), cholesterol, HDL-cholesterol, uric acid and lactate) and urinary urea were measured by enzymatic methods (Randox Laboratories, Crumlin, UK). Insulin and glucagon were assessed by radioimmunoassay (Millipore, Billerica, MA, USA). Plasma apolipoprotein B (apoBtot) and apolipoprotein B48 (apoB48) were measured by ELISA using kits from Shibayagi, Shibukawa, Japan and R & D Systems LTD, Abingdon, UK Plasma and urinary fructose concentrations and plasma 6,6-^2^H_2_-glucose isotopic enrichment were measured by gas chromatography-mass spectrometry (GC-MS). Two-point-three micromolar 1,2,3 ^13^C_3_ d-fructose was added to 250 mL plasma or urine as an internal standard. Plasma or urine samples were thereafter deproteinized using the ZnSO4-Ba(OH)2 method [20], partially purified over anion- and cation-exchange resins and derivatized with acetic anhydride and pyridine. Samples were then dried under a stream of nitrogen and resuspended in 60 μL ethyl acetate, and 1 μL was analyzed by GC-MS (Agilent Technologies, Santa Clara, CA, USA) in electron impact mode, with selected monitoring of *m*/*z* 275 and 277. The fructose concentration in samples was determined from the ratio of *m*/*z* 277 to *m*/*z* 275 by means of an unlabeled pure fructose standard curve. Plasma 6,6-^2^H_2_ glucose enrichment was measured on pentaacetyl-derivates, suing GC-MS in chemical ionization mode with selective monitoring of *m*/*z* 333 and *m*/*z* 331. Plasma ^13^C-lactate enrichments were measured on lactic *n*-propylamide heptafluorobutyrates, with selective monitoring of *m*/*z* 331 and *m*/*z* 328. Plasma ^13^C-glucose isotopic enrichment was measured by gas-chromatography-isotope ratio mass spectrometry (GC-C-IRMS), as described [8]. Plasma lipoprotein subfractions were separated by ultracentrifugation, and fractions S_f_ > 400 (chylomicrons) and S_f_ 20–400 (VLDL and chylomicron remnants) were isolated. Fatty acid methyl esters (FAMEs) from chylomicron- and VLDL-TG were isolated, and their ^13^C enrichment was measured by gas chromatography-isotope ratio mass spectrometry (Thermo Electron, Bremen, Germany), as described [21]. Tricosanoic acid methyl ester was used as an isotopic enrichment standard, and a quality-control sample (certified standard of eicosanoic acid FAME; Department of Geological Sciences, Indiana University, Bloomington, IN, USA) was run with each set of samples.

### 2.5. Calculations

Glucose rate of appearance (GRa) was calculated with 6,6-^2^H_2_-glucose as:

GRa (mg/min) = *F* + {[p × V × BW × (G_(*t*1)_/2) + (G_(*t*2)_/2)] × [(E_(*t*1)_/2 − E_(*t*2)_/2)]}/[(E_(*t*1)_/2 + E_(*t*2)_/2) × (*t*2 − *t*1)] (1)

where *F* = 6,6-^2^H_2_-glucose infusion rate (mg/min), p = pool fraction, set at 0.65, BW = body weight (kg), V = glucose distribution volume, set at 0.2, G = glucose concentration (mg/L), E = 6,6-^2^H_2_-glucose isotopic enrichment (mol% excess) and *t* = time of collection (min) [22]. Parameters were set assuming that kinetics for ^13^C-glucose appearance in blood after ingestion of a ^13^C-labelled glucose load apply to the ingestion of a ^13^C-labelled fructose load, as well [23].

Gluconeogenesis from fructose (GNGf) was calculated as:

GNGf (mg/min) = {GRa × [(^13^CG_(*t*1)_ + ^13^CG_(*t*2)_)/2] + p × V × [(G_(*t*1)_+G_(*t*2)_)/2] × [(^13^CG_(*t*2)_ − ^13^CG_(*t*1)_1)/(*t*2 − *t*1)]}/^13^C-fructose (2)

where ^13^CG = plasma ^13^C-glucose isotopic enrichment and ^13^C-fructose = meal ^13^C-fructose isotopic enrichment (at% excess).

Fructose oxidation (Fox) was calculated as:

Fox (mg/min) = 180 × (^13^CO_2(*t*x)_/^13^C-fructose_(*t*x)_) × (VCO2_(*t*x)_)/(22.29 × 6 × 0.8) (3)

where ^13^CO_2_ = breath CO_2_ isotopic enrichment (atom% excess), VCO_2_ = total CO_2_ production (L/min) and *tx* = time of collection; 22.29 mL CO_2_ was assumed to correspond to 1 mmol CO_2_; six mmol CO_2_ correspond to 1 mmol = 180 mg fructose; 0.8 is the recovery factor of ^13^CO_2_ in breath.

Non-oxidative fructose disposal (NOFD) was calculated as:

NOFD (mg/360 min) = (ingested fructose (g)) − (fructose oxidation, cumulated between 120 and 480 min (g)) (3)

The amount of ^13^C-glucose and ^13^C-lactate remaining in blood 6 hours after meal ingestion was low (<2 g) and was neglected in this calculation.

Net substrate oxidations and energy expenditure (EE) were calculated from standard indirect calorimetry equations [19], assuming that total nitrogen excretion was equal to (urinary urea nitrogen excretion)/0.85 [24].

For each meal, diet-induced thermogenesis was calculated as:

diet-induced thermogenesis (%) = 100 × (postprandial EE (kcal/360 min)) − (pre-prandial EE (kcal/min × 360 min))/(energy content of the meal kcal) (4)

### 2.6. Statistical Analysis

Results are expressed as mean ± SEM. The normality of data and differences was checked with Shapiro-Wilk tests for all parameters analyzed. Non-normally distributed data (glucagon, TG, VLDL-TG) were log-transformed before statistical analysis. All variables measured at different time points after ingestion of test-meals were reduced to a single value by calculating their average or cumulated postprandial values (gluconeogenesis from fructose, fructose oxidation, glycogen synthesis, net substrate oxidation, energy expenditure) or their cumulated incremental area under the curve values (iAUC: plasma concentrations of metabolites or hormones) over the 360-min postprandial period. Between test-meals, comparisons were performed on these single postprandial values by ANOVA followed by *pots hoc* paired *t*-tests with Bonferroni’s correction. A difference was considered significant when the Bonferroni-corrected *p*-value was <0.05. All statistical analyses were performed using STATA version 10 (Stata Corp, College Station, USA).

## 3. Results

### 3.1. Anthropometric Variables and Fasting Parameters

Fasting plasma glucose and insulin concentrations, body weight and body composition after three days on a controlled, weight maintenance diet were similar before each test meal (Table 2).

**Table 2:** Fasting parameters.

	ProLip	Fr	Fr + G
Plasma glucose (mmol/L)	4.74 ± 0.15	4.79 ± 0.08	4.67 ± 0.10
Plasma insulin (pmol/L)	54.2 ± 5.6	57.4 ± 4.7	54.3 ± 3.6
Body weight (kg)	63.4 ± 2.4	63.7 ± 2.4	63.6 ± 2.4
Lean body mass (kg)	51.1 ± 2.4	50.0 ± 2.5	50.4 ± 2.5
Fat mass (kg)	12.3 ± 1.1	13.7 ± 0.8	13.0 ± 1.0

All values are mean ± SEM; *n* = 8 subjects.

### 3.2. Effects on Carbohydrate Metabolism

**Figure 2:**
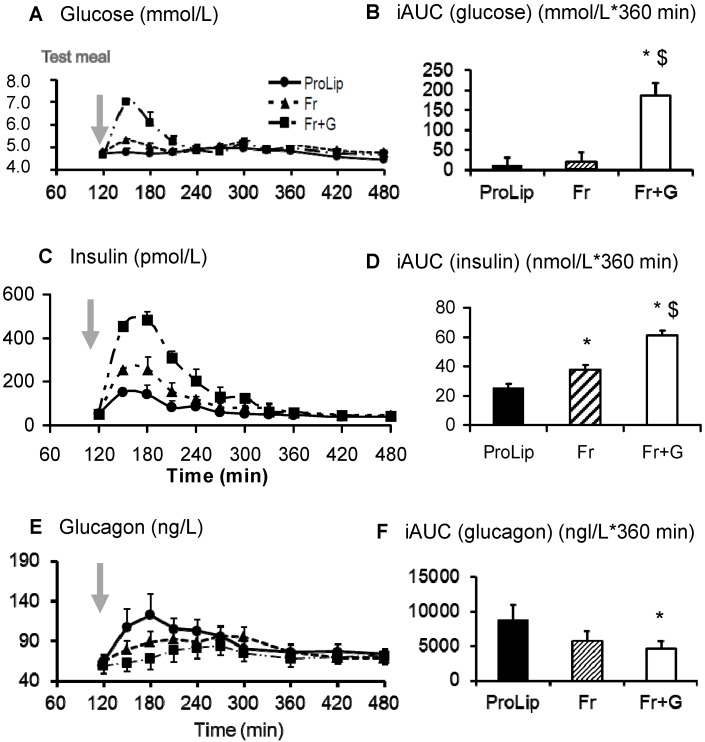
Plasma glucose, insulin and glucagon responses to meal ingestion. The time course for plasma glucose (**A**), plasma insulin (**C**), plasma glucagon concentrations and their corresponding incremental area under the curve (iAUC) values (**B**, **D** and **F**) (mean ± SEM, *n* = 8). Test meals were given at *t* = 120 min. iAUC were compared by *t*-tests with Bonferroni’s correction. Glucagon was log-transformed before statistical analysis. ProLip, lipid and protein; Fr, lipid, protein and fructose; Fr + G, lipid, protein, fructose and glucose. * *p* < 0.05 *vs*. ProLip; ^$^ *p* < 0.05 *vs*. Fr.

After ingestion of Fr + G, plasma glucose and insulin increased rapidly, peaking after 150 min, and, thereafter, declined progressively. Plasma glucagon concentrations were significantly decreased (*p* = 0.033) compared to ProLip (Figure 2).

**Figure 3:**
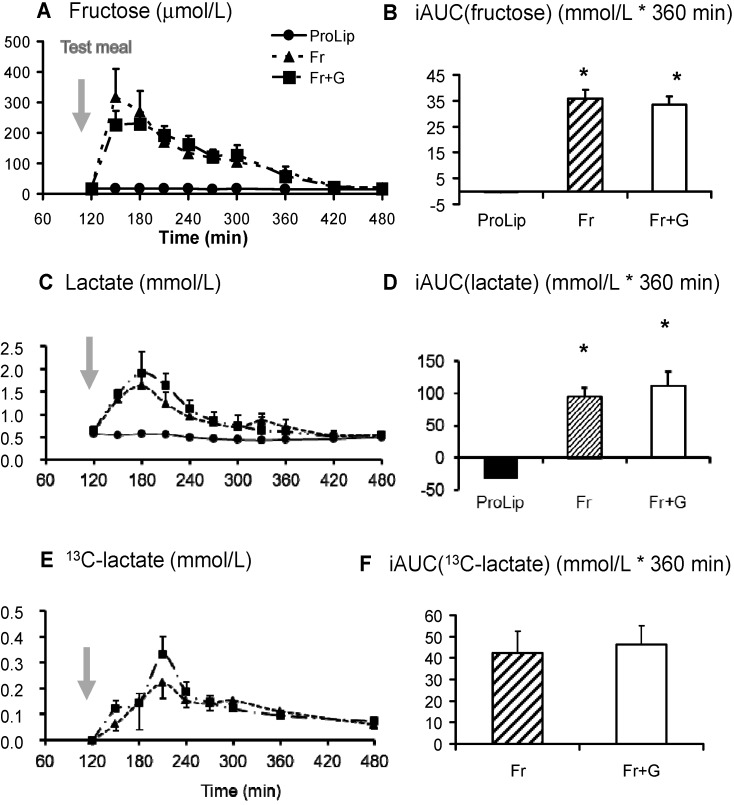
Plasma fructose, lactate and ^13^C-lactate responses to test meal ingestion. Time course for plasma fructose (**A**), lactate (**C**) and ^13^C-lactate concentrations (**E**) and their corresponding iAUC (**B**, **D** and **F**) (*n* = 8). * *p* < 0.05 *vs*. ProLip; ^$^ *p* < 0.05 *vs*. Fr.

Plasma fructose concentrations increased to an average peak value of 231 ± 37 μmol/L at 180 min and decreased rapidly thereafter (Figure 3). Plasma lactate and ^13^C-lactate concentrations also increased significantly and showed the same time-course as glucose (Figure 3). Plasma ^13^C-lactate enrichment averaged 0.15% mol% excess. Over the six hours after meal ingestion, since the enrichment of fructose in the meal was set at 1 mol% excess, this indicates that fructose contributed to approximately 15% of the total lactate production.

After Fr, the increase in plasma glucose and insulin concentrations was markedly blunted compared to Fr + G (Figure 2). In contrast, the increase in plasma fructose was of a similar magnitude as Fr + G. Plasma lactate and glucagon concentrations and plasma ^13^C-lactate isotopic enrichment were also not significantly different than after Fr + G (Figure 3). After ProLip, plasma glucose, fructose, insulin and lactate concentrations were minimally altered, but the rise in the plasma glucagon concentration was significantly higher than after Fr + G (Figure 2 and Figure 3).

**Figure 4:**
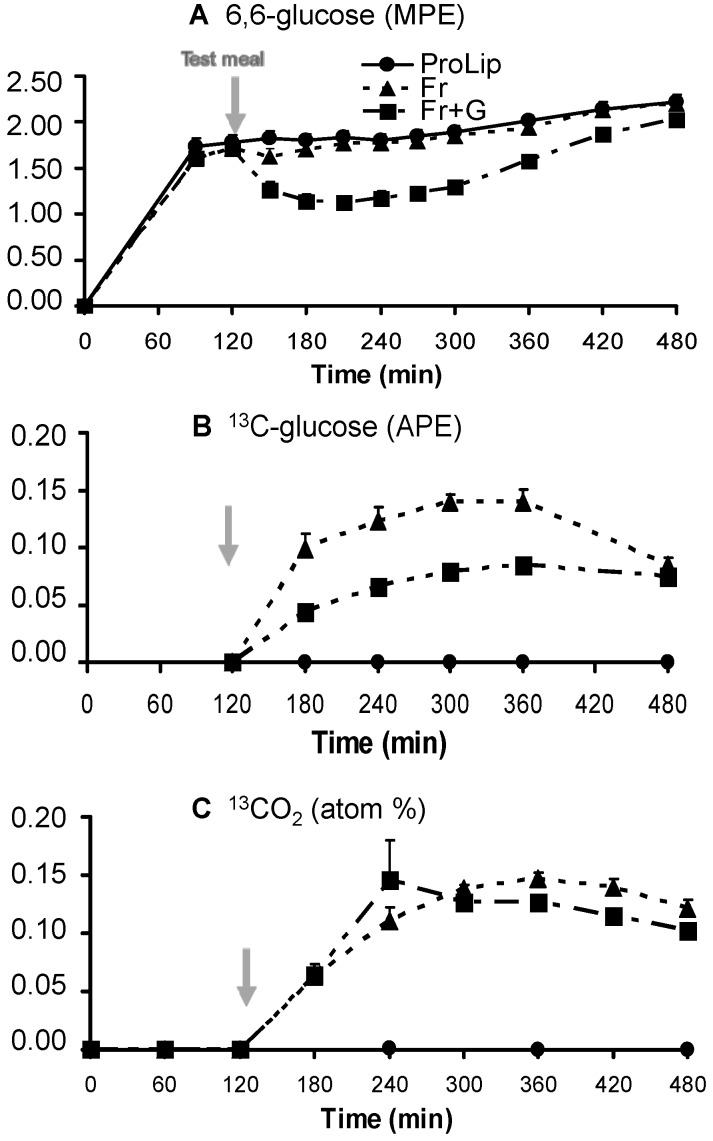
Plasma 6,6-^2^H_2_-glucose (**A**), plasma ^13^C-glucose (**B**) and breath ^13^CO_2_ (**C**) isotopic enrichments. (Mean ± SEM, *n* = 8). Test meals were given at *t* = 120 min. MPE: molar percent excess; APE: atom percent excess.

The release of fructose carbons as glucose in the systemic circulation were calculated from plasma 6,6-^2^H_2_- and ^13^C-glucose isotopic enrichments (shown in Figure 4), which allowed calculating GRa and GNGf, respectively, while the total oxidation of fructose was calculated from breath ^13^CO_2_ isotopic enrichment (Figure 4) and total CO_2_ production.

Over the 6 h following Fr + G ingestion, 33% of the ingested fructose was oxidized to CO_2_, and the remaining 67% was disposed of non-oxidatively (Table 3). Ingestion of Fr + G increased GRa from 2.09 ± 0.12 mg/kg/min in the basal condition to a mean postprandial value of 3.07 ± 0.12 mg/kg/min. With Fr, total GRa, cumulated over six hours postprandial, was lower, but GNGf, cumulated over the same period, was higher than with Fr + G. Cumulated fructose oxidation was also significantly higher and corresponded to 37% of ingested fructose, while non-oxidative fructose disposal was lower (Table 3). With ProLip, GRa was only marginally increased (basal: 2.09 mg/kg/min *vs*. average postprandial 2.22 mg/kg/min, NS) and, when cumulated over six hours postprandial, was comparable with that observed with Fr (Table 3).

**Table 3:** Total glucose rate of appearance (GRa, *n* = 8), fructose oxidation, total net carbohydrate oxidation (total carbo. ox., *n* = 6), gluconeogenesis from fructose (GNGf, *n* = 8) and non-oxidative fructose disposal (NOFD, *n* = 6) cumulated over six hours after the ingestion of test meals. * *p* < 0.05 *vs*. ProLip, ^$^ *p* < 0.05 *vs*. Fr.

	ProLip	Fr	Fr + G
GRa (g/360 min)	50.98 ± 2.47	53.78 ± 2.20	69.86 ± 2.51 *^,$^
Fructose oxidation (g/360 min)		12.64 ± 0.67	11.30 ± 0.74 ^$^
Total carbo. ox. (g/360 min)	25.92 ± 1.93	22.16 ± 1.91	25.87 ± 4.73
GNGf (g/360 min)		9.03 ± 0.15	6.54 ± 0.17 ^$^
NOFD (g/6 h)		21.78 ± 0.96	22.90 ± 0.97 ^$^

### 3.3. Effects on Lipid Metabolism

**Figure 5:**
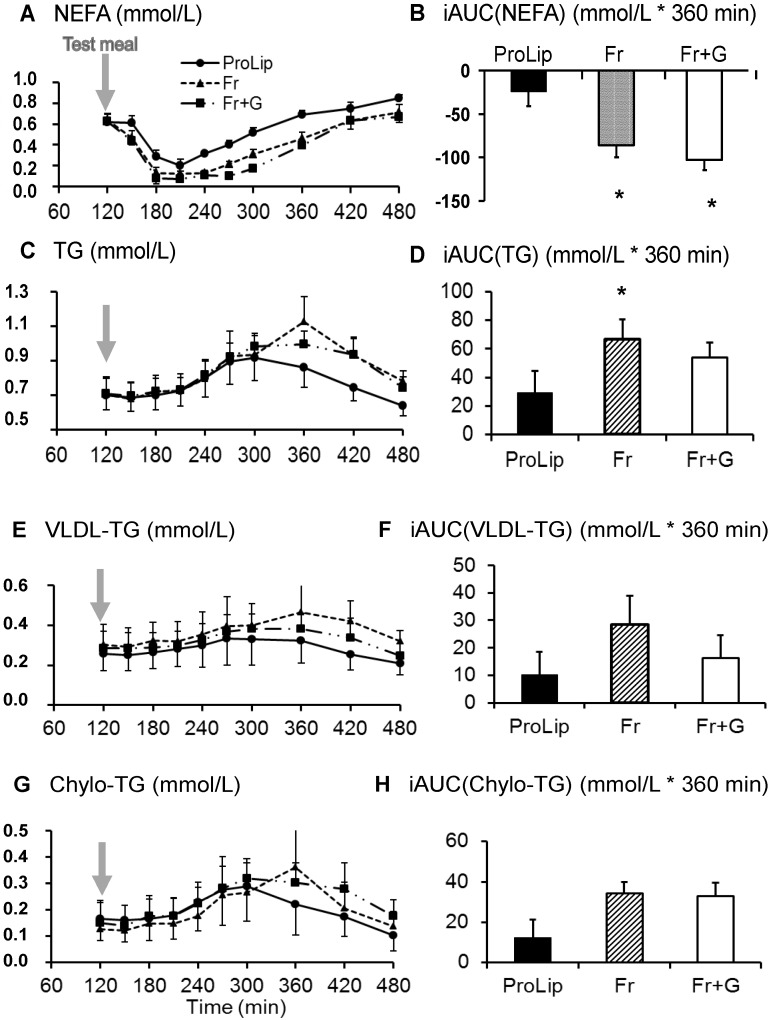
Plasma NEFA (**A**), total TG (**C**), VLDL-TG (**E**) and chylomicron-TG (**G**) responses to test meal ingestion and their corresponding iAUC (**B**, **D**, **F** and **H**) (*n* = 8). * *p* < 0.05 *vs*. ProLip, ^$^ *p* < 0.05 *vs*. Fr.

**Figure 6:**
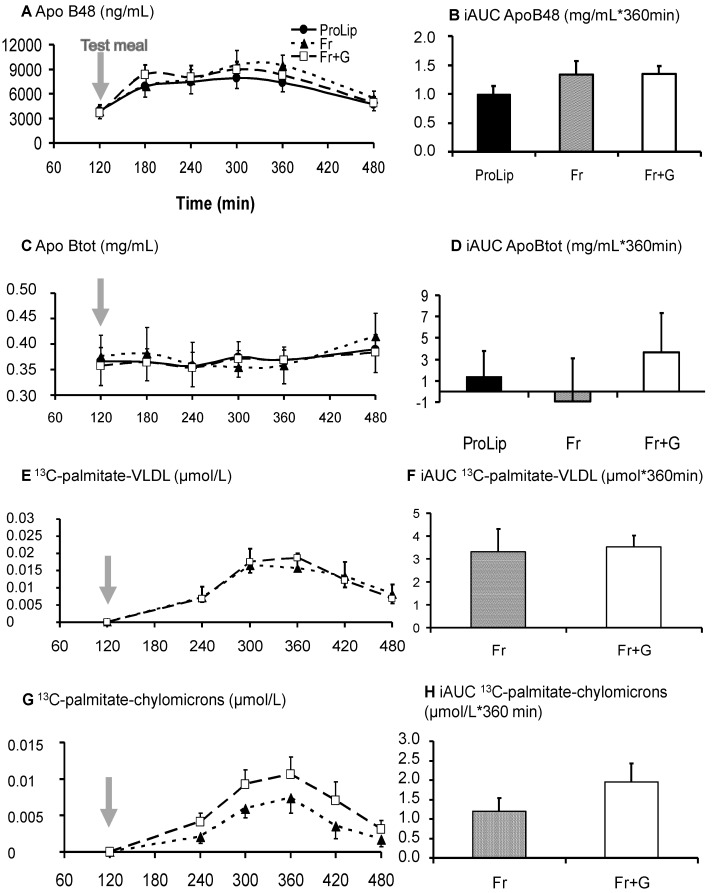
Plasma ApoB48 (**A**), ApoBtot (**B**), ^13^C-palmitate-VLDL (**E**), ^13^C-palmitate-chylomicrons (**G**) (*n* = 8). Additionally, their corresponding iAUC (**B**, **D**, **F** and **H**) (*n* = 8). * *p* < 0.05 *vs*. ProLip, ^$^ *p* < 0.05 *vs*. Fr.

With Fr + G, TG, VLDL-TG, chylomicron-TG and apoB48 concentrations and ^13^C-palmitate isotopic enrichment in chylomicrons and in VLDL-TG, all increased progressively to peak at around four hours after meal ingestion (Figure 5 and Figure 6). Plasma apoBtot concentrations did not change after meal ingestion, however. Plasma NEFA decreased transiently after ingestion of the meal and, thereafter, progressively increased. Postprandial net lipid oxidation amounted to 15.0 ± 4.1 g/360 min. With Fr, plasma TG, VLDL-TG and chylomicron-TG plasma apoB48 and plasma apoBtot responses were not significantly different from Fr + G. ^13^C-palmitate isotopic enrichment in VLDL-TG was also not different, but ^13^C-palmitate enrichment in chylomicron-TG tended to be lower with Fr than with Fr + G (*p* = 0.08; Figure 6). Total net lipid oxidation accounted for 16.5 ± 5.3 g/360 min.

With ProLip, postprandial plasma total TG concentrations were lower than with Fr (*p* < 0.05), but were not significantly different from Fr + G. VLDL-TG, chylomicron-TG and plasma ApoB48 concentrations did not show any significant differences with Fr + G or Fr (Figure 5 and Figure 6). Net lipid oxidation amounted to 18.0 ± 5.6 g/360 min and was not significantly different from Fr or Fr + G.

### 3.4. Effects on Energy Expenditure and Diet-Induced Thermogenesis

Basal energy expenditure was similar for all three test meals: ProLip: 1.05 ± 0.08 kcal/min; Fr: 1.07 ± 0.08 kcal/min; Fr + G: 1.00 ± 0.06 kcal/min. Postprandial energy expenditure, averaged over a 360-min postprandial period, tended to be higher with Fr (1.15 ± 0.08 kcal/min, *p* = 0.06), but not with Fr + G (1.13 ± 0.07 kcal/min, *p* = 0.31, NS) compared to ProLip (1.09 ± 0.08 kcal/min,). Diet-induced thermogenesis did not significantly differ between the three meals (Fr + G: 9% ± 1%; Fr: 7% ± 2%; ProLip: 7% ± 1%, NS).

## 4. Discussion

The measurements obtained in this study provide novel insights into the metabolic fate of fructose when ingested as part of a mixed meal. The experimental design involved the ingestion of three different protein- and fat-based drinks, one containing ^13^C- labelled fructose and unlabeled glucose in equimolar amounts (Fr + G), one containing the same amount of ^13^C fructose, but without glucose (Fr), and one containing neither fructose nor glucose (ProLip). Since the amount of ^13^C fructose ingested was the same in Fr + G and Fr, comparing the appearance of ^13^C in breath CO_2_ and in plasma metabolites provided information on how the co-ingestion of glucose impacted the metabolic fate of fructose; in addition, since the amount of lipid was the same in all three meals, comparing the postprandial plasma TG responses provided information on how glucose and fructose impacted on postprandial lipemia.

### 4.1. Metabolic Fate of Fructose Ingested together with Equimolar Amounts of Glucose

When both ^13^C-fructose and glucose were present in the drink, about one third of the ingested ^13^C fructose carbons were recovered as breath ^13^CO_2_ over 360 min after ingestion of Fr + G. This represents both the oxidation of fructose in splanchnic organs and extrahepatic oxidation of some newly synthesized glucose and lactate released into the systemic circulation. The methods used in this study do not allow us to evaluate the relative importance of each pathway to total fructose oxidation. Assuming that exogenous fructose was completely absorbed from the gut after 360 min, one can nonetheless conclude that about 70% of the ingested fructose load was deposited into the body energy stores, either as glycogen or as TG in the liver or in extrahepatic tissues.

Ingestion of the mixed meal containing both labelled fructose and unlabeled glucose caused a small transient increase in plasma fructose concentration, but a significant incorporation of ^13^C in plasma glucose, lactate, VLDL-TG and chylomicron-TG. This is consistent with an extensive fructose uptake in splanchnic organs and its conversion into glucose, lactic acid and lipids [24]. The contribution of ^13^C-glucose synthetized from ^13^C-fructose to whole body glucose fluxes could be quantified with the simultaneous use of 6,6-^2^H_2_ glucose and accounted for 19.0% ± 1.5% of ingested fructose over the 360 min postprandial period. This is an underestimate of total hepatic gluconeogenesis from fructose, however, since part of the newly formed glucose was likely to be retained within intrahepatic glycogen. Plasma ^13^C-lactate isotopic enrichments indicate that about 15% of circulating lactate was produced from fructose, presumably in the gut and liver. However, the amount of lactate actually produced from fructose could not be quantified, since lactate turnover was not measured.

There was also a significant incorporation of labelled carbons into both VLDL- and chylomicron-palmitate. The bulk of ^13^C-palmitate was detected in the VLDL-TG fraction, corroborating earlier reports that fructose ingestion stimulates *de novo* lipogenesis in liver cells [1,15,25]. In addition, we observed an incorporation of ^13^C in chylomicron-palmitate, indicating that some fructose was converted into TG within enterocytes, as reported in high fructose-fed rodents [12], and more recently in humans [14,26]. The concentration of ^13^C-palmitate in chylomicron-TG was about one third of that observed in VLDL-TG, but this may underestimate the relative contribution of the gut to total *de novo* lipogenesis, since chylomicrons have a shorter half-life and, hence, a higher turnover rate than VLDL [27]. The total amount of fructose converted into fat during these experiments could not be quantified, because we used a semi-quantitative isotopic approach, thus we could not actually measure the isotopic enrichment of intrahepatic acetyl-coA from which fatty acids are synthesized; we did not quantitatively assess the VLDL- and chylomicron-TG secretion rate, nor whether newly-synthesized fatty acids were being stored as intrahepatic fat [28]. Furthermore, ^13^C-palmitate concentrations measured in plasma lipoproteins reflected only the conversion of ^13^C-fructose into lipids, but not the contribution of unlabeled glucose carbons.

### 4.2. Modulation of Fructose Metabolism by Glucose

Postprandial plasma fructose concentrations were identical when the test meal included fructose alone and both fructose and glucose. This suggests that glucose in the gut lumen did not significantly alter splanchnic fructose extraction. This may appear surprising, since fructose absorption is known to be quantitatively limited when pure fructose is ingested alone [29], but to be facilitated by glucose [28]. The fructose load ingested with the test-meals was relatively small and, hence, may not have exceeded gut fructose absorptive capacity; furthermore, it is possible that fructose transport had been upregulated as a consequence of having consumed a 20% sucrose diet during the several days prior to the experiments [30].

More labelled fructose carbons were recovered as breath ^13^CO_2_ when fructose was added alone to the protein and fat meal than with glucose. In addition, a larger portion of labelled fructose carbons was released as plasma glucose, while plasma lactate and ^13^C-lactate concentrations were not altered significantly. We can therefore conclude that glucose inhibited gluconeogenesis from fructose, while, at the same time, enhancing the storage of fructose carbons, presumably as hepatic glycogen. However, this difference represented only 1.5 g or about 5% of ingested fructose.

Postprandial VLDL-^13^C-palmitate, plasma apoBtot and apoB48 concentrations were not significantly different when fructose was ingested alone or together with glucose. This suggests that co-ingestion of glucose had not grossly altered the proportion of ingested fructose, which was converted into fat in the liver. In contrast, postprandial the chylomicron-^13^C-palmitate response tended to be higher when glucose was co-ingested with fructose, suggesting that intestinal *de novo* lipogenesis may have been enhanced by glucose [31].

### 4.3. Effects of Fructose and Glucose on Dietary Lipid Handling

After the ingestion of a protein and lipid meal devoid of carbohydrate, total-, chylomicron- and VLDL-TG and plasma apoB48 concentrations all increased progressively to reach a maximum after about three hours and progressively decreased thereafter. When fructose alone or both fructose and glucose were added to the meal, the initial TG response was not altered, but the rises in total, chylomicrons and VLDL-TG were extended for an additional hour; and the integrated TG response tended to be larger. The difference only attained statistical significance when comparing the effects of the protein and lipid meal with and without fructose alone. Plasma apoBtot and apoB48 responses were not altered, suggesting that the effect of fructose may be due to an increased *de novo* lipogenesis rather than to an impaired extra-hepatic clearance of TG-rich lipoproteins. Taken together, our results corroborate the finding that fructose added to a meal enhances postprandial blood lipids [32] and further indicate that co-ingestion of glucose does not further enhance this effect.

### 4.4. Limitations

Our study has some limitations that need to be outlined. First, our experimental design involved the administration of three test meals with the same protein and lipid content, but differing in their fructose and glucose content. This was done as an attempt to assess the specific effects of fructose and its interaction with glucose. Thus, it resulted in comparing three energy-unbalanced test meals, and it remains possible that the effects on fructose metabolism that we attributed to glucose co-ingestion were in part due to the higher energy content of the Fr + G meal. We also considered the possibility that the different macronutrient composition of test meals may have altered the gastric emptying rate, which may have altered the rate of fructose carbon metabolism. The postprandial time course and peak values for plasma fructose concentration were not significantly different after the Fr and the Fr + G meal, which suggests that the rate of gut fructose absorption and the rate of gastric emptying were not altered by the addition of glucose to the Fr meal. However, it remains possible that sugars may have altered gastric emptying after the Fr and Fr + G meals compared to Pro-Lip and that this may have affected postprandial plasma TG to some extent. Second, we documented how glucose co-ingestion modulated the disposal of ingested fructose, but did not attempt to evaluate whether the reverse is also true, *i.e*., whether the presence of fructose in a meal alters glucose metabolism, as well. Third, we selected liquid test meals made of cream and purified whey protein, but devoid of starch and fiber for practical purposes, and cannot exclude that the metabolism of fructose ingested with a solid mixed meal may differ from that observed with a liquid, starch- and fiber-free meal. Finally, this study included a small number of participants and may have failed to detect differences in TG-rich lipoprotein ^13^C-palmitate concentrations between Fr and Fr + G due to low statistical power.

## 5. Conclusions

Fructose included in a liquid meal containing lipid, protein and glucose is essentially extracted and metabolized in splanchnic organs, where it is quickly converted into glucose, lactate and fatty acids, thus corroborating rodent studies [12,13] and some recent human reports [14,26]. These findings are consistent with previous isotope studies in humans [21]. Our data further indicate that both the liver and the gut synthesize fat [14,24]. About two-thirds of ingested fructose remained temporarily stored in the body after 360 min, presumably as glycogen and lipids; present methods do not allow for the assessment of the relative contribution of these two processes. Co-ingestion of glucose and fructose, compared to fructose alone, significantly decreased fructose oxidation and gluconeogenesis and increased fructose carbon storage. In contrast, it did not enhance postprandial plasma lipid concentrations and did not alter the recovery of ^13^C carbons into plasma chylomicron- and VLDL-TG.
